# Enhanced Blood Compatibility of Metallocene Polyethylene Subjected to Hydrochloric Acid Treatment for Cardiovascular Implants

**DOI:** 10.1155/2014/963149

**Published:** 2014-05-15

**Authors:** Saravana Kumar Jaganathan, Hemanth Mohandas, Gunalan Sivakumar, Palaniappan Kasi, Theertha Sudheer, Sruthi Avineri Veetil, Selvakumar Murugesan, Eko Supriyanto

**Affiliations:** ^1^IJN-UTM Cardiovascular Engineering Centre, Faculty of Biosciences and Medical Engineering, Universiti Teknologi Malaysia, 81310 Johor Bahru, Malaysia; ^2^Department of Research and Development, PSNA College of Engineering and Technology, Kothandaraman Nagar, Dindigul, Tamil Nadu 624 622, India; ^3^Rubber Technology Centre, Indian Institute of Technology, Kharagpur, West Bengal 721302, India

## Abstract

Blood compatibility of metallocene polyethylene (mPE) was investigated after modifying the surface using hydrochloric acid. Contact angle of the mPE exposed to HCl poses a decrease in its value which indicates increasing wettability and better blood compatibility. Surface of mPE analyzed by using FTIR revealed no significant changes in its functional groups after treatment. Furthermore, scanning electron microscope images supported the increasing wettability through the modifications like pit formations and etching on the acid rendered surface. To evaluate the effect of acid treatment on the coagulation cascade, prothrombin time (PT) and activated partial thromboplastin time (APTT) were measured. Both PT and APTT were delayed significantly (*P* < 0.05) after 60 min exposure implying improved blood compatibility of the surfaces. Hemolysis assay of the treated surface showed a remarkable decrease in the percentage of lysis of red blood cells when compared with untreated surface. Moreover, platelet adhesion assay demonstrated that HCl exposed surfaces deter the attachment of platelets and thereby reduce the chances of activation of blood coagulation cascade. These results confirmed the enhanced blood compatibility of mPE after HCl exposure which can be utilized for cardiovascular implants like artificial vascular prostheses, implants, and various blood contacting devices.

## 1. Introduction

The viewpoints of polymer science have become more understandable in the past 60–70 years and so the use and applications of polymers in medicine has similarly gained momentum. Some of the popular polymers that gained their place in the world of the biomaterial due to their nontoxic nature and diverse mechanical properties are polyethylene, polyurethanes, silicone rubber, and synthetic biodegradable polymers such as poly (*α*-esters) and polyanhydrides [1–3]. But there are problems associated with implantable polymer medical device which include device associated infection [4], thrombus formation [5], and tissue sequestration of the implant [6]. New protocols or designs aimed at designing compatible polymers circumventing these detrimental conditions are being investigated specially in the case of cardiovascular biomaterials.

Cardiovascular biomaterials (CB) broadly fall into the three main types, namely, biological substances, metals, and polymers. One of the most versatile classes of CB is polymers. Physical and mechanical properties of polymers can be varied to suit the need of cardiovascular applications with ease. Some applications of polymers such as CB are vascular grafts, catheter, tubing, shunt, artificial heart valves structure, oxygenator, blood bags, and dialysis membranes. The statistics of the last decade by Markets and Markets showed that the orthopedic biomaterial market had the highest market share, but now the market is shifting towards cardiovascular biomaterials due to the increasing number of cardiac patients around the world. The global biomaterial market is estimated to reach $88.4 billion by 2017 from $44 billion in 2012 out of which 34.5% will be contributed by the cardiovascular biomaterials segment [7]. Research is still continuing in developing CB with minimum side effects and good biocompatibility.

The two most important factors that affect the interactions are surface topology and chemistry [8–10]. As a matter of fact, a number of researches are carried on the study and modification of certain polymeric surfaces to improve biocompatibility [11–15]. Various surface treatments carried out for improved biocompatibility are grafting copolymerization [11], plasma treatment [12], UV and laser irradiation [13], dielectric discharge [14], and microwave plasma irradiation [15].

Modern developments in polymer technology like metallocene single-site catalyst produced a new class of polyolefins with better performance properties like enhanced toughness, sealability, clarity, and elasticity [16]. Metallocene consists of two cyclopentadienyl anions (Cp,) which are bound to a metal center (M) with the oxidation state II, resulting in a general formula M (C_5_H_5_)_2_ [16, 17]. One among the polymers developed through metallocene technology is metallocene polyethylene (mPE). mPE finds applications in disposable bags, storage bottles, blood bags, and syringe tubes. Although mPE has an excellent permeability to oxygen and acts as a barrier towards ammonia and water, still mPE lacks blood compatibility to make it ideal for blood contacting cardiovascular implants [18].

Recently microwave assisted surface improvement of mPE was investigated for its improved blood compatibility. Microwave treatment resulted in enhanced blood compatibility by increasing the surface roughness and decreasing the blood clotting time [18]. In this work, an attempt is made for improving blood compatibility of mPE using commonly available acid like hydrochloric acid (HCl). HCl has been used for surface modification of metallic biomaterial like titanium [19]. A thorough literature survey indicates that no report on the effect of hydrochloric acid on this mPE surface has been documented. Hence, this study aims to observe the surface characteristic and blood compatibility variations of mPE after exposing it to HCl.

## 2. Materials and Methods

### 2.1. Ethics Statement

All protocols pertaining to the use of blood were approved by the Institutional Ethical Committee at PSNA College of Engineering and Technology, Dindigul. Blood was withdrawn by venipuncture from aspirin-free healthy adult human donors and anticoagulated with tri-sodium citrate in a 9 : 1 volumetric ratio. Freshly prepared platelet rich plasma (PRP) was obtained from the Dindigul Blood Bank, Dindigul, India.

### 2.2. Sample Preparation and Acid Treatment

Two mPE films of dimensions 10 cm × 10 cm were cut into samples sizes of 1 cm × 2 cm. Each sample was washed with 70% ethanol and distilled water before HCl treatment. The acid treatment is done for 30 min and 60 min. Petri dish is cleaned and dried and 6–8 mL of 11.32 M hydrochloric acid is poured into the dish. The dried samples are treated in the acid for 30 min and 60 min. Later the samples are taken and placed on another petri dish to dry. While preparing the samples for blood compatibility tests, the samples are placed in a beaker of physiological saline and are kept in rotary shaker overnight at 37°C to wash away the acid present on the surface of the polymer.

### 2.3. Surface Characterization Tests

Surface of the polymer can be analyzed with the help of surface characterization tests. Here, the wettability, chemical composition, and microstructure of the polymers were analyzed using contact angle, FTIR, and SEM, respectively.

#### 2.3.1. Contact Angle Measurement

The hydrophilicity studies of the polymer were performed with the help of Data physics DCAT 11. The contact angles were recorded and analyzed for untreated samples and 30 min and 60 min treated samples (*n* = 3).

#### 2.3.2. Attenuated Total Reflectance Fourier Transfer Infrared Spectroscopy (ATR-FTIR)

ATR-FTIR equipment NEXUS-870 model spectrophotometer equipped with extended beam splitter, two light sources, and middle band MCT detectors with various sampling options was used to analyze the chemical compositions or functional groups present within the polymer. Three samples, namely, untreated and 30 min and 60 min HCl-treated samples, were analyzed in this study.

#### 2.3.3. Scanning Electron Microscope

Surface microstructure of the samples can be visualized and studied in detail with the help of scanning electron microscope images. The SEM used to analyze the polymeric samples is JEOL JSM 5800 SEM with OXFORD ISI 300 EDS X-ray Microanalysis System. The samples underwent gold sputtering and were then studied in SEM at 1500x.

### 2.4. Coagulation Assays

A polymer-induced abnormality in the blood clotting cascade was studied with the help of coagulation assays. Endpoint of these assays is the onset of a fibrin clot when the platelet poor plasma comes in contact with the acid treated and untreated sample surfaces. Hemolysis assay estimated the damage to the red blood cells. Platelet adhesion assay was performed to study the interaction between the platelet and the surface of the polymer.

#### 2.4.1. Prothrombin Time

Prothrombin time is a useful indicator to assess the interdiction of extrinsic pathway. Platelet poor plasma (PPP) (100 *μ*L at 37°C) was applied on the surface of the untreated and treated substrates along with NaCl-thromboplastin (Factor III, 100 mL, Sigma) containing Ca^2+^ ions. The time taken for the onset of fibrin clot was estimated with the aid of a stopwatch and a steel hook (*n* = 3) [18].

#### 2.4.2. Activated Partial Thromboplastin Time (APTT)

APTT is widely used to assess the ability of the blood to coagulate through the intrinsic pathway and to assess the effect of biomaterial on possible delay of the process. Platelet poor plasma (100 *μ*L at 37°C) is preincubated with the substrates at 37°C and then activated by addition of rabbit brain cephalin (100 *μ*L 37°C). The samples were incubated at 37°C for 5 min and then incubated with calcium chloride (0.025 M). Addition of CaCl_2_ initiates the clotting process. The time taken from the addition of CaCl_2_ till the clot is formed is recorded as the activated partial thromboplastin time (APTT) (*n* = 3) [18].

#### 2.4.3. Hemolysis Assay

The untreated and the treated samples (30 min and 60 min) were equilibrated with physiologic saline (0.9% w/v; 37°C, 30 min) and then incubated with 3 mL aliquots of citrated blood diluted with saline (4 : 5 ratios by volume). The mixture of blood and distilled water was prepared in the ratio 4 : 5 by volume to cause complete hemolysis which was taken as the positive control. The negative control is the physiological saline solution which produces no coloration. The samples were incubated in their respective mixtures (60 min, 37°C). These mixtures were then centrifuged and the absorbance of the clear supernatant was measured at 542 nm. The absorbance of positive control was normalized to 100% and the absorbance of different samples was expressed as a percentage of hemolysis when compared with their positive control (*n* = 3) [18].

#### 2.4.4. Platelet Adhesion Assay

The samples were treated with HCl for 60 min and were incubated with physiological saline (0.9% w/v; 37°C, 30 min) and kept on the rotary shaker for an hour to cleanse them of the residues on the surface of the polymer. The untreated and treated samples were each immersed in 1 mL fresh PRP and were incubated at 37°C for an hour. PRP was decanted off and the membranes were rinsed with physiologic saline and dried. Later, the samples were viewed through the microscope (*n* = 3). The surface of the polymer was photographed and the number of platelets was counted on a region with 40x magnification [20].

### 2.5. Statistical Analyses

All experiments were performed three times independently. One-way ANOVA was performed to find statistical significance. All quantitative experiment results are expressed as mean ± SD.

## 3. Results

### 3.1. Contact Angle Measurements

The hydrophilicity behavior of the polymer is summarized in Table 1. The mean contact angle of untreated sample was found to be 86.12°. This is much greater when compared with the treated samples especially with 60 min acid treated sample. The mean contact angles of 30 and 60 min treated samples are 70.18° and 54.07°, respectively (Table 1). Decrease in contact angle implies the increased wettability and hydrophilicity of the mPE polymer.

### 3.2. Attenuated Total Reflectance Fourier Transform Infrared Spectroscopy (ATR-FTIR)

**Figure 1:**
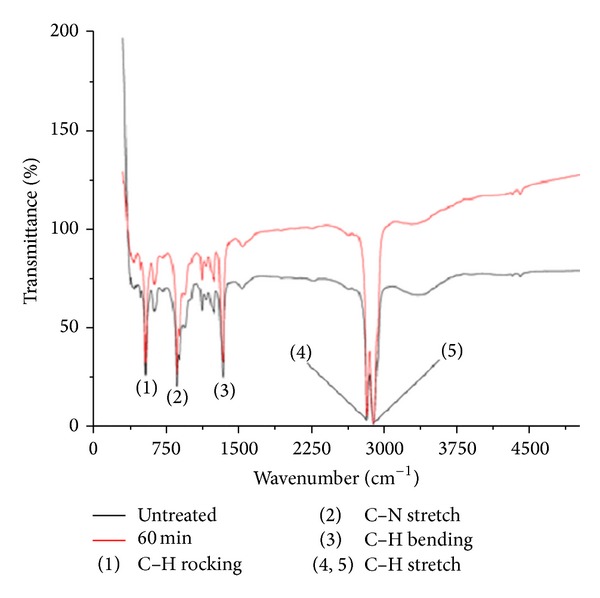
A representative FTIR spectra of untreated and 60 min HCl-treated metallocene polyethylene.

FTIR studies were done and carefully analyzed for the chemical composition of untreated and treated samples (Figure 1). There were no changes in the functional groups between the treated and the untreated ones. There were similar peaks observed at wavelengths 2850 cm^−1^ and 2930 cm^−1^ belonging to the alkane group (C–H stretch). Peaks were also noticed at 1470 cm^−1^ (C–H bending) and finally at 725 cm^−1^ (C–H rocking), belonging to the alkane family but with differences in their structures. A peak was also ascertained at 1020 cm^−1^ belonging to the aliphatic amines group (C–N Stretch). However, the intensity of the peaks associated with the treated surface was found to vary with untreated one. The intensity of absorption was increased with respect to all peaks in the acid treated mPE. These observations indicate that there are alterations in the surface morphology.

### 3.3. Scanning Electron Microscope

**Figure 2:**
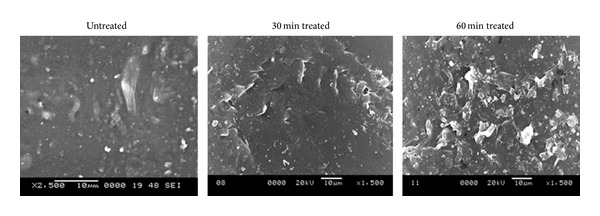
Representative SEM micrographs of untreated and HCl-treated metallocene polyethylene.

Surface of the polymers was visualized and morphological studies of the polymer samples were made (Figure 2). On observing the SEM image of 30 min treated sample, it can be observed that the surface of the treated samples has been affected by acid exposure. A few number of pits formation were also noticed. Also, the size of the pits seems to increase in the case of 60 min acid treated sample. A larger surface disorientation and increasing roughness were noticed in 60 min treated sample.

### 3.4. Coagulation Assay

#### 3.4.1. Prothrombin Time (PT) and Activated Partial Thromboplastin Time (APTT)

**Figure 3:**
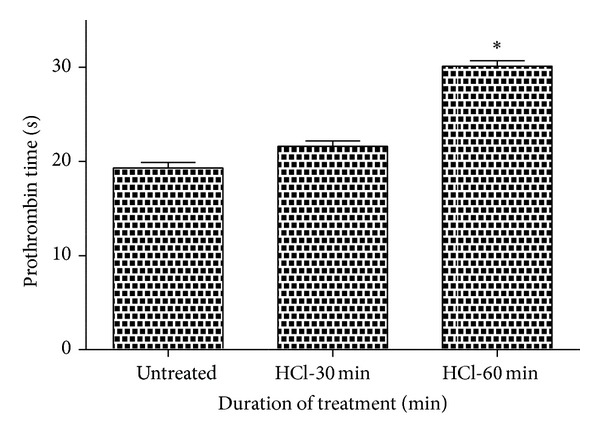
Comparison of prothrombin time (PT) of untreated and HCl-treated metallocene polyethylene (*n* = 3). Values shown are mean ± SD and ∗  indicates difference in the mean is significant with *P* < 0.05.

**Figure 4:**
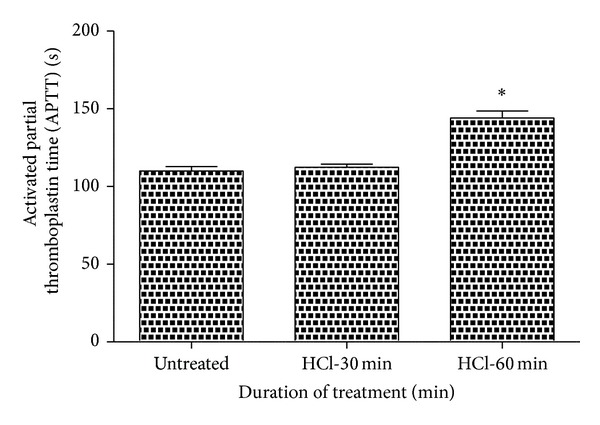
Comparison of activated partial thromboplastin time (APPT) of untreated and HCl-treated metallocene polyethylene (*n* = 3). Values shown are mean ± SD and ∗ indicates differences in the mean are significant (*P* < 0.05).

Prothrombin time and activated partial thromboplastin time were carried out on the three mentioned samples, namely, untreated and 30 min and 60 min HCl treated. Their results were summarized in Figures 3 and 4, respectively. Both PT and APTT showed an increase in their value for the treated samples than the untreated. Mean PT of untreated sample was found to be 19.3 s, whereas 30 and 60 min HCl exposed samples displayed 21.6 s and 30.1 s, respectively. Similarly, mean APTT was found to be 110 s, 112.3 s, and 144 s for the untreated and 30 min and 60 min treated mPE. The mean of PT and APTT performed without biomaterial surfaces yielded 16.7 s and 96.2 s, respectively. Statistical analysis of the untreated sample with the treated ones using one-way ANOVA indicated significant differences (*P* < 0.05) between them for both PT and APTT times after 60 min exposure.

#### 3.4.2. Hemolysis Assay

**Figure 5:**
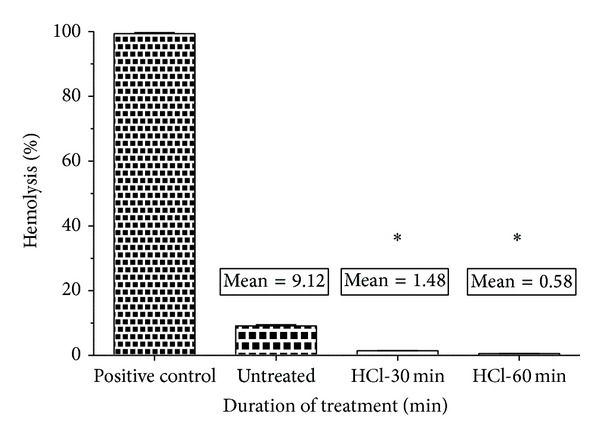
Comparison of percentage of hemolysis of untreated and HCl-treated metallocene polyethylene (*n* = 3). Values shown are mean ± SD and ∗ indicates differences in the mean are significant (*P* < 0.05).

The hemolysis test was carried out on the treated samples and untreated sample in order to study the effect of polymer surface on red blood cells (RBC). Mean percentage of hemolysis seemed to decrease in the case of treated samples (1.487% and 0.587% for 30 min and 60 min HCl-treated samples) compared with the untreated (9.123%) mPE, insinuating lesser damage and interaction between the treated samples and RBC (Figure 5). Statistical analysis of the untreated sample with the treated one (percentage of hemolysis) using one-way ANOVA indicated significant differences (*P* < 0.05) between them after 30 and 60 min treatment.

#### 3.4.3. Platelet Adhesion Assay

**Figure 6:**
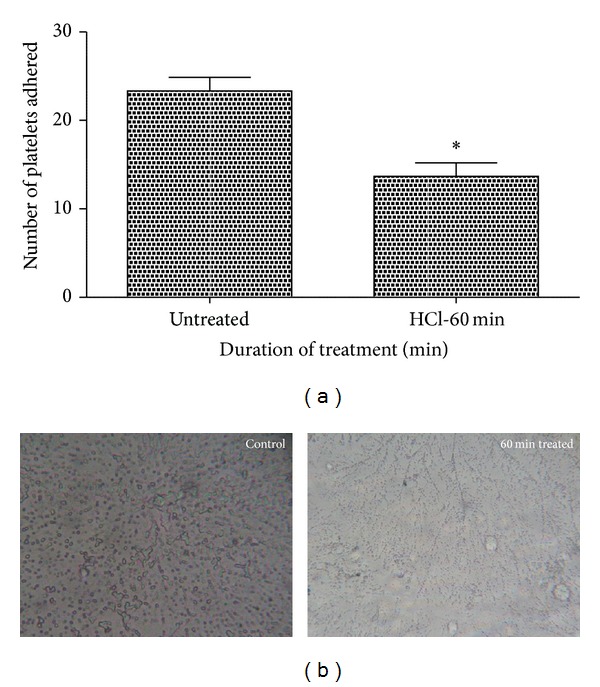
Platelet adhesion assay of untreated and HCl-treated metallocene polyethylene (*n* = 3). Figure 6(a) indicates the number of platelets adhered on the untreated and 60 min HCl exposed surface and values are expressed as mean ± SD. ∗ Differences in the means are significant with *P* < 0.05. Figure 6(b) shows photomicrograph of untreated and 60 min HCl-treated mPE at 40x magnification.

The number of platelets adhered on the surface of treated polymers seems to decrease to nearly half of the number of platelets which was found on the untreated sample. A maximum of 25 platelets was found on the surface of the untreated samples, whereas the number of platelets reduced to maximum of 14 platelets on 60 min treated samples (Figure 6(a)). Statistical analysis of the untreated sample with the treated one (number of platelets adhered) using one-way ANOVA indicated significant differences (*P* < 0.05) between them after 60 min treatment. The photograph of the polymer surface also proves the decrease in the number of platelets found on the treated compared to the untreated polymeric surfaces (Figure 6(b)).

## 4. Discussion

One of the major problems associated with cardiac biomaterial used for blood contacting devices is the compatibility of the materials with the blood. To circumvent the above, various strategies were adopted to modify the surface in order to improve the compatibility of the material. Among them, modification of the surface using strong acids is one of the ways to improve the compatibility [19, 21] and in this paper HCl-assisted changes in the surface and blood compatibility were deciphered.

Contact angle measurement indicated that the angles for the treated polymers were decreased when compared with untreated surface. Decreasing contact angle indicates higher degree of wettability and hence improved biocompatibility [22]. A decreasing contact angle is attributed to chemical or morphological changes associated with the polymer [23]. To ascertain the changes associated with functional groups, FTIR analysis was performed. There were no qualitative changes in the functional groups of the acid treated polymer. However the intensity of the peaks (2850 cm^−1^, 2930 cm^−1^, 1470 cm^−1^, 1020 cm^−1,^ and 725 cm^−1^) was different between the untreated and acid exposed surfaces signifying the alterations in the surface. From the above results it is indicated that the functional groups of the polymer are not affected by the acid treatment which motivated us to study the morphological changes associated with the surface after treatment.

One of the researches explained that the roughness has a vital role in controlling the thrombogenicity and it depicted that catheters with increased roughness were found to be less thrombogenic than smooth surfaces [24]. Similarly another research conducted on hydrophilic surface pointed out that increased surface roughness resulted in decreased platelet adhesion [25, 26]. Recently concluded research observed that when the roughness was increased by a TiO_2_/Ta_2_O_5_ nanofilm on NiTi alloy, platelet activation, adhesion, and hemolysis were greatly reduced [27]. Furthermore researchers have illustrated the increase in wettability accompanied by the increasing in surface roughness and associated decrease in the contact angle [22, 23, 28]. SEM images of acid rendered surfaces indicated an increase in surface roughness and distortions on the treated polymers. A large number of pit formations were also observed in the acid treated polymers. It is hypothesized that this surface modification by HCl associated with increased surface roughness may have a putative role in promoting the biocompatibility of the cardiovascular devices like catheter of short-term use. The most immediate reaction of blood with the biomaterial surface is plasma protein adsorption and activation of blood components which may be retarded by the increased surface roughness of mPE polymer due to this treatment. From the above observations, the surface of the polymer has morphological changes to a great extent, but to confirm its use in cardiovascular implants of long-term use, the blood compatibility of the mPE has to be evaluated.

The preliminary events that take place between the surrounding and the cardiovascular implants interface are the formation of thrombosis mediated by the surface interactions with adsorbed proteins (intrinsic pathway) or through the release of tissue factor from the damaged cells at injury site (extrinsic pathway) [29]. Adsorbed surface proteins form a complex composed of collagen, high molecular weight kininogen (HMWK), prekallikrein, and factor XII. Inactive precursors (clotting factors) change confirmation and are converted into active enzymes via a biochemical cascade resulting in platelet activation (with the aid of additional cofactors) [21]. In order to study how surface modified and untreated mPE interacts with blood, various blood compatibility tests were carried out. Initially to assess the effect of surface modification, prothrombin time (PT) and activated partial thromboplastin time (APTT) were evaluated to understand the tissue-implant interaction mediated through extrinsic and intrinsic pathway, respectively [18, 19]. Both PT and APTT were delayed significantly and this prompts us to consider modified mPE as a potent player in cardiovascular biomaterials. The persuasiveness of the study was carried out with the help of hemolysis assay and platelet adhesion studies. The results of these assays further insinuated that modified mPE resulted in lesser damage to RBC and also reduced adhesion of platelets on the surface. These two characteristics are vital for any biomaterial which has to be considered for cardiovascular applications of long-term use. Hence, modified mPE with increased surface roughness, altered wettability, and better hemocompatibility may be the essential traits that can be exploited for construction of long-term devices like vascular prosthesis and other blood contacting devices.

In conclusion, hydrochloric acid treatment of mPE was investigated for its effect on blood compatibility. Contact angle analysis of the treated sample signified a fall in the contact angle indicating an increase in the wettability of the samples. There were no remarkable qualitative changes in the functional groups as revealed by FTIR studies. SEM images of treated samples showed that acid treated surface is occupied with pits formations. Blood coagulation assays like PT and APTT revealed that there is a delay in the clotting mechanism on the surface of treated samples. The results of hemolysis assay depicted lesser damage to red blood cells (RBC). Platelet adhesion assay revealed that the number of platelets adhering on the surface of the treated polymer was significantly less than that on the untreated surface. As a whole, the hydrochloric acid treatment of the mPE shows good pattern of the results to be used as an implant in vascular grafts and other medical implants especially in the case of cardiovascular implants due to its better blood compatibility.

## Figures and Tables

**Table 1:** Contact angle measurement of the mPE before and after HCl treatment.

S. Number	Sample	Average contact angle in degrees*
1	Untreated mPE	86.12 ± 2.16
2	mPE treated with HCl (30 min)	70.18 ± 1.85
3	mPE treated with HCl (60 min)	54.07 ± 1.12

Data represents mean ± S.D; *Mean differences are significant at *P* < 0.05, (*n* = 3).
